# Platelet microRNAs inhibit primary tumor growth via broad modulation of tumor cell mRNA expression in ectopic pancreatic cancer in mice

**DOI:** 10.1371/journal.pone.0261633

**Published:** 2021-12-22

**Authors:** Jeremy G. T. Wurtzel, Sophia Lazar, Sonali Sikder, Kathy Q. Cai, Igor Astsaturov, Andrew S. Weyrich, Jesse W. Rowley, Lawrence E. Goldfinger

**Affiliations:** 1 Division of Hematology, Department of Medicine, Cardeza Center for Hemostasis, Thrombosis, and Vascular Biology, Cardeza Foundation for Hematologic Research, Sidney Kimmel Medical College, Thomas Jefferson University, Philadelphia, PA, United States of America; 2 Molecular Therapeutics Program and The Marvin & Concetta Greenberg Pancreatic Cancer Institute, Fox Chase Cancer Center, Philadelphia, PA, United States of America; 3 Cancer Biology Program and Histopathology Facility, Institute for Cancer Research, Fox Chase Cancer Center, Philadelphia, PA, United States of America; 4 Molecular Medicine Program, Pathology Division, Department of Internal Medicine, University of Utah, Salt Lake City, UT, United States of America; 5 Molecular Medicine Program, Pulmonary Division, Department of Internal Medicine, University of Utah, Salt Lake City, UT, United States of America; Children’s National Hospital, UNITED STATES

## Abstract

We investigated the contributions of platelet microRNAs (miRNAs) to the rate of growth and regulation of gene expression in primary ectopic tumors using mouse models. We previously identified an inhibitory role for platelets in solid tumor growth, mediated by tumor infiltration of platelet microvesicles (microparticles) which are enriched in platelet-derived miRNAs. To investigate the specific roles of platelet miRNAs in tumor growth models, we implanted pancreatic ductal adenocarcinoma cells as a bolus into mice with megakaryocyte-/platelet-specific depletion of mature miRNAs. We observed an ~50% increase in the rate of growth of ectopic primary tumors in these mice compared to controls including at early stages, associated with reduced apoptosis in the tumors, in particular in tumor cells associated with platelet microvesicles—which were depleted of platelet-enriched miRNAs—demonstrating a specific role for platelet miRNAs in modulation of primary tumor growth. Differential expression RNA sequencing of tumor cells isolated from advanced primary tumors revealed a broad cohort of mRNAs modulated in the tumor cells as a function of host platelet miRNAs. Altered genes comprised 548 up-regulated transcripts and 43 down-regulated transcripts, mostly mRNAs altogether spanning a variety of growth signaling pathways–notably pathways related to epithelial-mesenchymal transition—in tumor cells from platelet miRNA-deleted mice compared with those from control mice. Tumors in platelet miRNA-depleted mice showed more sarcomatoid growth and more advanced tumor grade, indicating roles for host platelet miRNAs in tumor plasticity. We further validated increased protein expression of selected genes associated with increased cognate mRNAs in the tumors due to platelet miRNA depletion in the host animals, providing proof of principle of widespread effects of platelet miRNAs on tumor cell functional gene expression in primary tumors *in vivo*. Together, these data demonstrate that platelet-derived miRNAs modulate solid tumor growth *in vivo* by broad-spectrum restructuring of the tumor cell transcriptome.

## Introduction

Platelets have been associated with tumor progression and metastatic dissemination through platelet-tumor cell interactions and regulation of tumor angiogenesis [1–6]. A relationship between platelets and tumor progression was recognized many years ago, when Gasic, Gasic and Stewart showed in animal models that induced thrombocytopenia blocked tumor metastasis [1]. Subsequent studies have linked platelet count, platelet activation state, or expression of platelet proteins with tumor incidence, progression and metastasis [7, 8]. Platelet-tumor cell interactions may contribute to tumor progression in several ways, including enhancing cancer-related coagulation, and providing a tumor cell “shroud” to shield them from the immune system [9]. The presence of cancer increases platelet production, which has been associated with poorer outcomes in multiple cancers [10–12]. Platelets can modulate proliferation of human and murine cancer cells in a manner that does not require platelet-tumor contact [12]. However, the platelet-cancer axis still remains unsolved and is an area of active investigation.

Platelets and other cells release microvesicles into the plasma in response to receptor agonists and shear stress [13]. As high as 70–90% of plasma-borne microvesicles are platelet-derived microvesicles (PMV) [14, 15]. PMV release increases in individuals bearing solid tumors, but roles of PMV in cancer progression are incompletely understood [16, 17]. PMV are enriched in platelet microRNAs (miRNAs), a small cohort of which are present at high copy number, accounting for the bulk of plasma miRNAs [18–21]. MiRNAs are genomically encoded short non-coding RNAs that function to suppress gene expression by hybridizing to complementary mRNAs and blocking protein translation through multiple mechanisms [22, 23]. Purified PMV are able to transfer at least some miRNA content to cells following co-incubation *in vitro*, and regulate gene expression [24–27]. Several miRNAs enriched in PMV, including miR-27a, miR-24, miR-155, miR-195, let-7a/b and miR-223, target both tumor suppressor genes and oncogenes, in multiple cancer types, and have been identified as diagnostic and prognostic markers of malignancy, and implicated in therapy resistance [28–33]. For example, miR-24 and miR-223 have each been associated with multiple cancer types including pancreatic cancers, and targets include both tumor suppressors and oncogenes, with new targets and modes of action continually being discovered [27, 31, 34–40]. Thus, the functional outcomes of miRNA activity in cancers remain to be fully explained, and represent a ripe area for research and development of therapeutic strategies.

Our previous studies using ectopic solid tumor growth models in mice demonstrated that platelet-derived miRNAs can be transferred via infiltrating PMV to the tumor cells, associated with suppression of *in vivo* tumor growth, in part via transfer of platelet-derived miR-24 [41]. PMV, like those of all cells, harbor many types of molecules, many of which have been shown to have effects on tumor cell function across various model systems [42, 43]. PMV surfaces resemble those of activated platelets, and thereby have the opportunity to interact with heterologous cells including tumor cells and cells in the tumor microenvironment, in a similar fashion as activated platelets [44]. In addition, as described above, platelets themselves have direct and indirect functions in cancer progression, and in this sense PMV may act in large part as extended platelet populations, as expansions of the activated platelet pool in terms of interactions with tumor cells and cells in the tumor microenvironment. Whereas PMV are established to infiltrate solid tumors in humans and mice, and transfer platelet-derived miRNAs to the tumor cells with growth modulatory effects, whether platelets behave in similar fashion in the context of solid tumors is not clear. Thus, the specific roles of platelet components in tumor progression require further elucidation. In this study, we sought to determine the specific contributions of platelet miRNAs to primary ectopic tumor growth and modulation of tumor cell gene expression in tumors using *in vivo* mouse models of ectopic pancreatic adenocarcinoma. Our results demonstrate that platelet miRNAs as a cohort are tumor suppressive via broad-spectrum restructuring of the tumor cell transcriptome.

## Materials and methods

### Animals and ethics

*Dicer1*^fl/fl^/*Pf4-Cre* and *Pf4-Cre* C57Bl/6 female mice were housed at 2–5 mice per cage in standard shoebox cages in a temperature- and humidity-controlled environment with a 12/12-hour light-dark cycle. Food and water were available *ad libitum*. Mice were randomly allocated to experimental groups. All tumor implantation was performed under isofluorane anesthesia, and all efforts were made to minimize suffering. Tumor resections were carried out post-mortem. This study was carried out in strict accordance with the recommendations in the Guide for the Care and Use of Laboratory Animals of the National Institutes of Health. The protocol was approved by the Institutional Animal Care and Use Committee at Thomas Jefferson University (protocol number 02232). Humane endpoints based on body weight loss, body condition scoring and tumor progression were in place. Mice were euthanized prior to a maximum allowed tumor burden of 600 mm^3^ tumor volume. Mice were euthanized with CO_2_ followed by cervical dislocation. No unexplained mortality occurred in these studies.

### Reagents and cell lines

Anti-Tenascin C antibodies were from Novus Biologicals (Centennial, CO, USA); anti-Cadherin 17 antibodies were from R&D Systems (Minneapolis, MN, USA); anti-Mucin 4, cleaved Caspase-3 and -E-cadherin antibodies were from Cell Signaling Technology (Danvers, MA, USA); anti-Cd41 antibodies were from BD Biosciences (San Jose, CA, USA); anti-Tissue Factor antibodies were from Sekisui Diagnostics (Burlington, MA, USA); anti-Vim and–pan-cytokeratin antibodies were from Abcam. Fluorophore-conjugated anti-Cd45, -Cd16, -Cd41 and–Cd3 antibodies were from Biolegend (San Diego, CA, USA). Fluorophore-conjugated secondary antibodies and pre-immune IgG were from Jackson (West Grove, PA, USA).

The KPC3 pancreatic adenocarcinoma cell line was derived from spontaneous pancreatic ductal adenocarcinomas in *LSL-Kras*^*G12D/+*^*;LSL-Trp53*^*R172H/+*^*;Pdx-1-Cre* mice on the C57Bl/6J background, by single cell sorting of resected tumors as described [45]. Cells were cultured in Dulbecco’s Modified Eagle’s Medium (DMEM) with 10% FBS in a humidified, 5% CO2 atmosphere at 37°C.

### Tumor implantation, measurements, resection and processing

For tumor allografts, suspensions of 1 x 10^6^ cells/200 mL of Hanks balanced salt solution were injected subcutaneously into the shaved flanks of 8-week-old mice. Tumors were measured with calipers, resected, processed, and analyzed as described [45, 46]. Volumes were calculated using the formula Volume = long axis x short axis^2^ x 0.52, as described previously [46, 47].

### Nanoparticle tracking

Nanoparticle tracking analysis was performed with a NanoSight NS300 fitted with a 488 nm laser (Malvern, Westborough, MA, USA). Filtered media or buffer (0.22 μm filter) was analyzed each time for background subtraction; typical counts were ~10^5^. Final particle counts were derived from averaging the final concentrations with background subtracted, for six scans per sample, 30 sec per scan. Addition of 0.1% Triton X-100 caused immediate disappearance of event counts, indicating that the particle counts represented membranous structures (results not shown).

### RNA isolation, cDNA preparation and high throughput sequencing

Tumors were resected from euthanized mice and separated from fat, skin and connective tissues. Resected tumors were rinsed in PBS, diced with a fresh razor blade, and suspended in PBS containing 1 mg/ml collagenase A for 45 min with gentle agitation at 37°C, in the continuous presence of 0.1 μg/mL actinomycin D to inhibit transcription. The tissue/cell suspensions were filtered through a 70-μm strainer. The filtered cell suspensions were centrifuged for 5 minutes at 1,300 rpm, the cell pellets were washed once with PBS, and incubated with anti-Epcam-Alexa647 for 60 min at 4°C, rinsed 3x in ice-cold PBS, and suspensions were kept cold and separated by single cell sorting using a FACS Aria IIμ cell sorter (BD, Franklin Lakes, NJ, USA) equipped with a 633 nm laser. Sorted tumor cells were pelleted by centrifugation at 4°C, frozen and kept at -80°C until RNA extraction in TRIzol, RNA precipitation with ethanol and resuspension in DEPC water. For platelet microvesicle qRT-PCR studies, murine platelets were isolated from freshly extracted anti-coagulated blood, resuspended in Tyrode’s buffer and stimulated to release microvesicles with 0.1 U/mL thrombin at 37°C with gentle stirring for 2 hours, followed by collection of microvesicles by centrifugation of platelet-depleted release fractions at 15,000x*g* for 90 min, as described [41, 48]. RNA extraction from platelet microvesicle fractions, first strand cDNA synthesis, and qRT-PCR for mature miRNAs, were prepared and performed as described [41, 48]. Single bands of predicted product size for each PCR reaction were confirmed by gel electrophoresis from all qRT-PCR samples. Gene expression levels relative to controls were determined using the 2^-ΔΔCt^ method with Gapdh as housekeeping gene. For RNA sequencing, tumor samples were prepared using Illumina TruSeq Stranded polyA mRNA library kit and sequenced single-end 50 cycles on an Illumina HiSeq. Reads were aligned with STAR [49] and DESeq2 used for differential expression [50]. Over-representation analysis was performed with the tool WebGestalt [51].

### Immunoblotting, immunocytochemistry and immunohistochemistry

Immunoblotting was carried out as described [41]. For immunohistochemistry, issues were fixed in 3.7% paraformaldehyde, embedded in paraffin, and 5 μm sections were collected on charged slides, immersed in xylene for 5 minutes and rehydrated. Slides were incubated with 0.4% pepsin at 37°C for 20 minutes, washed 4x in TBS, followed either by hematoxylin/eosin staining, or in some cases by 0.1% Triton X-100, washes, then incubated with primary antibodies at a dilution of 1:200 plus 1:100 dilution normal donkey serum, overnight at 4°C. The next day, sections were washed 4x, then 1 mg/ml solutions of fluorescein isothiocyanate (FITC)-, Cy3- or Cy5- conjugated antibodies were applied at a dilution of 1:100 (FITC) or 1:2000 (Cy3/5), for 1 h at room temperature, washed, and mounted with under coverslips with 80% glycerol/TBS. Images were acquired on an EVOS FL Auto microscope with a 10x or 60x/1.42 NA oil-immersion objective at room temperature with Pearl EVOS software, or a Zeiss LSM 780 microscope with a 63x/1.40 NA oil-immersion objective at room temperature utilizing a TCS SL confocal system running Zeiss ZEN 2011 SP7 FP3 (64bit) software. Post-acquisition image processing was performed using ImageJ and Adobe Photoshop. Operations included brightness/contrast adjustment to all pixels in the images, grouping of images, and automated particle counting.

### Statistics

Tumor volume, mass and histological scoring measurements were performed and analyzed by observers blinded to the host genotype. Statistical testing was done using GraphPad Prism software (Graphpad, La Jolla, CA). In some cases, data were normalized to starting measurements to control for baseline variation and statistical analysis was performed prior to normalization. *P* < 0.05 was considered statistically significant. Values are expressed as mean ± s.e.m. unless otherwise stated. Additional statistical testing was performed for RNA-seq analysis as outlined in the caption for S1 Table. Experiments were repeated at least 3 times unless otherwise stated.

## Results

### Platelet miRNAs suppress in vivo ectopic solid tumor growth from pancreatic cancer cells

**Fig 1:**
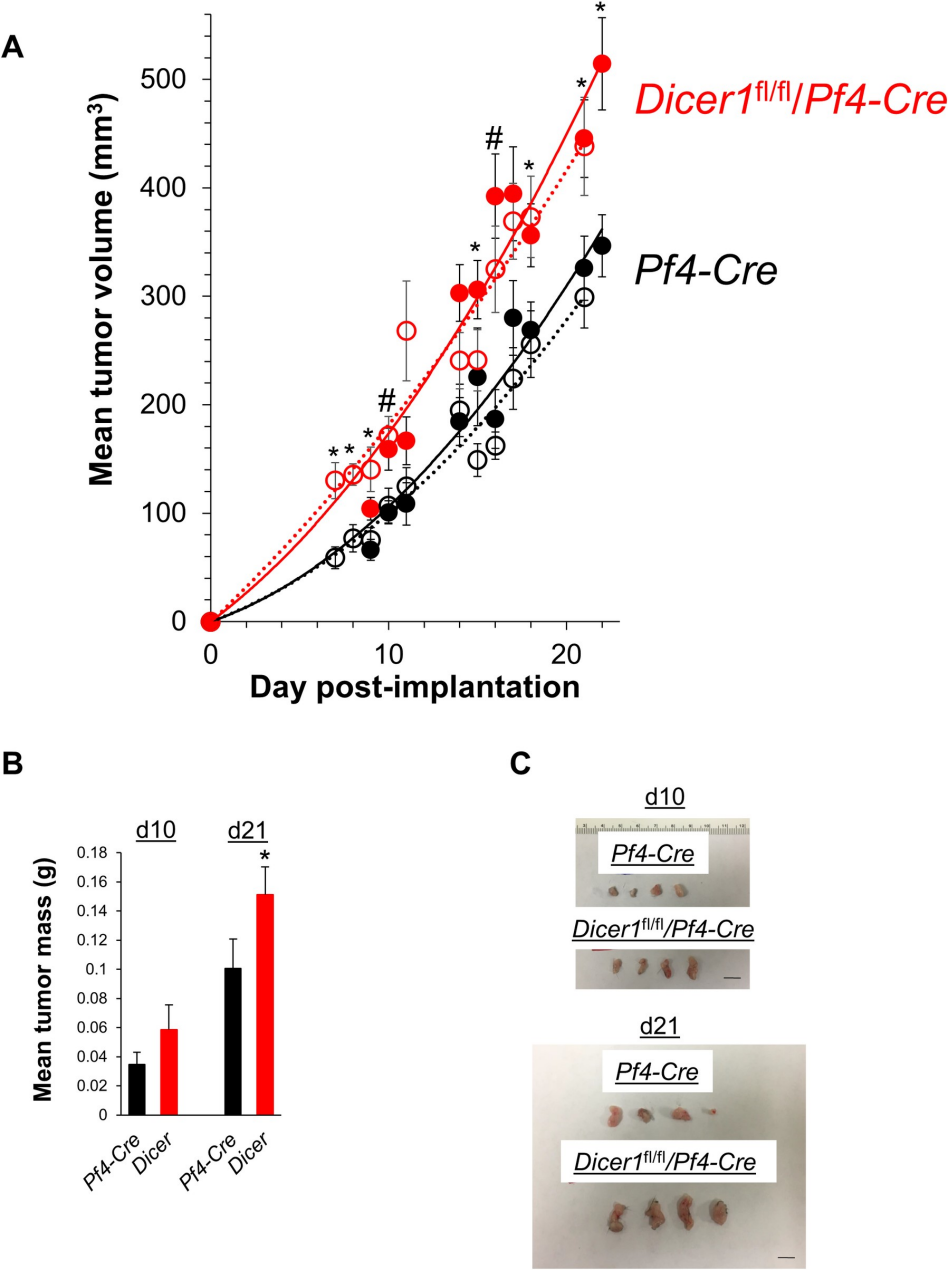
Platelet microRNAs suppress *in vivo* ectopic solid tumor growth in mice. **A** KPC ectopic primary tumor growth measured by calipers. Average volumes from twelve tumors per type per experiment are shown at each time point ± s.e.m. Solid and dotted line pairs represent independent experiments. Experiment 2 (solid lines) concluded at 22 days post-implantation, as shown. **B** Masses of primary tumors isolated at the indicated times (*n* = 12). Representative data are shown + s.e.m. from one of four independent experiments. **C** Representative resected tumors. Bar, 1 cm. *, *p* < 0.05; #, *p* < 0.02.

To investigate specific roles for platelet miRNAs in primary solid tumor growth *in vivo*, we implanted “KPC” murine pancreatic carcinoma cells into the flanks of *Dicer1*^fl/fl^/*Pf4*-Cre C57Bl/6 mice, which are depleted of miRNAs specifically in megakaryocytes/platelets due to selective Dicer1 deletion in megakaryocytes, or *Pf4*-Cre C57Bl/6 control mice [52]. The KPC tumor cell line was derived from spontaneous pancreatic ductal adenocarcinomas in KPC mice on the C57Bl/6 background—*e*.*g*., mice with *Pdx1-Cre*-driven pancreatic epithelial-specific expression of KRas^G12D^/p53^R172H^ which develop adenocarcinoma with 100% penetrance [45, 53]. We monitored ectopic *in vivo* tumor growth in the flanks of these mice by measuring tumor volumes daily with calipers beginning seven days after implantation when subcutaneous tumors could first be observed and measured, and continuing for three weeks. At the earliest time point of seven days post-implantation, ectopic KPC tumors implanted in *Dicer1*^fl/fl^/*Pf4*-Cre mice displayed a mean volume of 130.1 ± 16.7 mm^3^, compared with 58.9 ± 10.0 mm^3^ in control mice, a 2.2-fold increase in tumor volume in mice with platelet-specific loss of miRNAs at this initial time point (Fig 1A). In a repeat of this experiment, tumors were first detected at nine days after implantation, and the tumor volumes in *Pf4*-Cre and *Dicer1*^fl/fl^/*Pf4*-Cre mice were 66.1 ± 9.8 mm^3^ and 104.0 ± 10.5 mm^3^, respectively, at this first time point–a 1.57-fold increase in tumor volume in platelet miRNA-depleted mice. This trend of accelerated tumor growth against a background of miRNA-depleted host platelets continued over the experimental time frame, resulting in an overall 1.54 ± 0.07-fold mean daily increase in tumor volume from the *Dicer1*^fl/fl^/*Pf4*-Cre mice compared with control mice (Fig 1A). Tumor mass differences at d10 post-implantation showed a trend toward higher tumor mass in the *Dicer1*^fl/fl^/*Pf4*-Cre mice, but this difference was not significant. However, tumor mass was significantly increased by 1.50 ± 0.13-fold at d21 from the *Dicer1*^fl/fl^/*Pf4*-Cre mice compared with control mice, reflecting similar increases in tumor volumes (Fig 1B and 1C). These results demonstrate that depletion of miRNAs in platelets in the host mice was sufficient to increase the rate of ectopic pancreatic cancer cell tumor growth by ~1.5-fold in this model.

**Fig 2:**
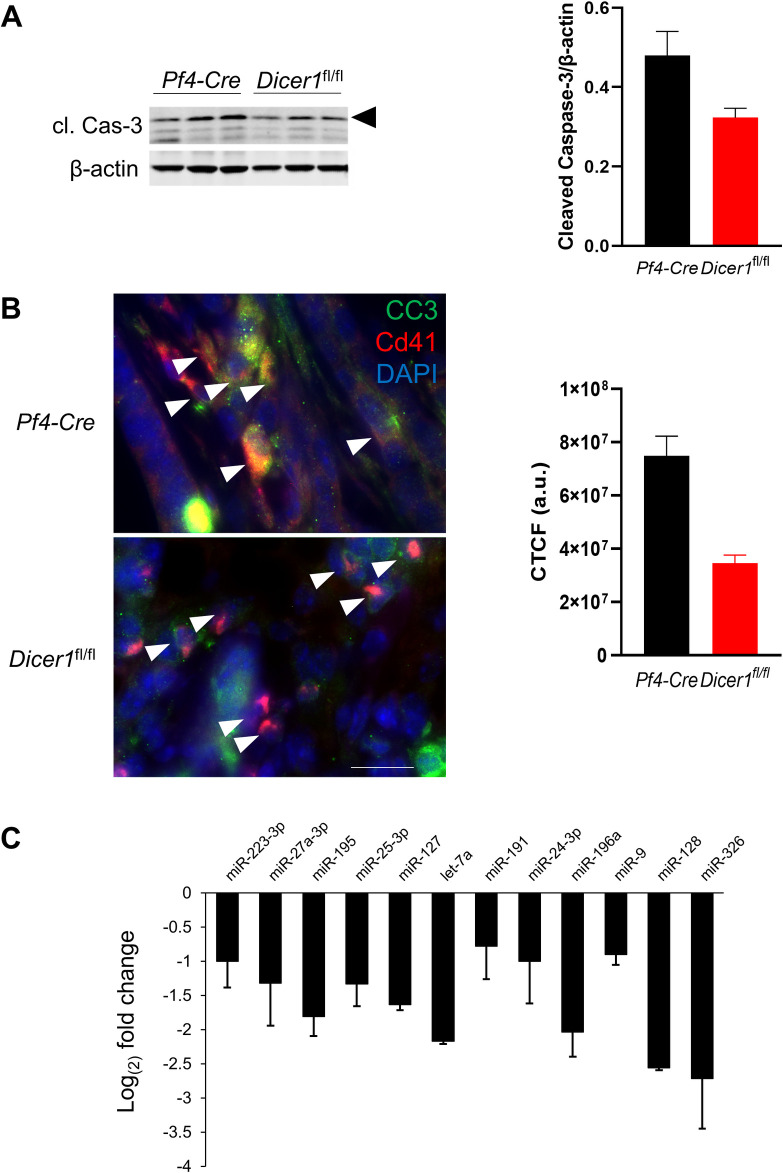
Platelet miRNA- and microvesicle-associated tumor cell apoptosis. **A** Representative western blots of cleaved Caspase-3 (cl. Cas-3, arrowhead) and β-actin in whole tumor lysates, three for each host animal genotype. Densitometric measurements of cleaved Caspase-3/β-actin ratios are shown below + s.e.m. *n* = 6. *, *p* < 0.05; #, *p* < 0.02. **B** Representative sections of KPC tumors resected from *Pf4-Cre* or *Dicer1*^fl/fl^/*Pf4*-Cre mice, immunostained with antibodies to cleaved Caspase-3 (CC3, green), Cd41 (red), and Epcam to identify and outline tumor cells (not shown), followed by DAPI (blue) to show nuclei. White arrowheads point to selected tumor cells with platelet- or platelet microvesicle-derived Cd41 staining. Bar, 10 μm. **C** Corrected total cellular fluorescence of CC3 staining, measured per cell as integrated density divided by cell area [56], in Epcam/Cd41 double-positive cells from 10 fields per tumor type, shown + s.e.m. At least 60 cells were analyzed per type. *p* < 0.03. **D** qRT-PCR for platelet-enriched mature miRNAs, in platelet-derived microvesicles. 1 x 10^8^ platelets from three mice of each genotype were isolated and stimulated to release microvesicles, which were collected and processed for RNA extraction and first strand cDNA synthesis as outlined in Methods. qRT-PCR results are shown as log_(2)_ fold change for each miRNA in samples from *Dicer1*^fl/fl^/*Pf4*-Cre mice compared to *Pf4-Cre* mice,—s.e.m. MiRNA species tested were the -5p arm unless otherwise indicated. *Dicer1*^fl/fl^ indicates *Dicer1*^fl/fl^/*Pf4*-Cre.

In previous studies in similar ectopic tumor models, we observed increased incidence of apoptosis, as indicated by enrichment of apoptotic effector cleaved Caspase-3, in primary tumor cells which were associated with platelet-derived microvesicles *in vivo*. We also reported a similar effect in tumor cells treated *in vitro* with platelet microvesicles, and this effect was dependent on platelet miRNAs [41]. Therefore, we tested a specific role for platelet miRNAs in tumor cell apoptosis *in vivo*, by comparing cleaved Caspase-3 enrichment in lysates of ectopic KPC tumors resected from *Dicer1*^fl/fl^/*Pf4*-Cre mice versus from control mice. Total cleaved Caspase-3 was significantly reduced in tumor lysates from the platelet miRNA-depleted mice compared with control mice (Fig 2A), indicating that platelet miRNA-dependent modulation of primary tumor growth results in part from modulation of progression to apoptosis in the tumor cells. To investigate direct roles for platelet-derived microvesicles and miRNAs in tumor cell apoptosis *in vivo*, we stained sections of resected tumors with Cd41 antibodies to identify tumor cells associated with platelet plasma membrane-derived material. We observed Cd41 staining indicative of platelet microvesicles, consistent with our previous identification of platelet microvesicle infiltration and tumor cell association *in vivo*, as well as putative expression of Cd41 in tumor cells, which has been shown to occur following tumor cell internalization of platelet microvesicles (Fig 2B) [41, 54]. These Cd41+ tumor cells showed strong staining for cleaved Caspase-3 in tumors from *Pf4*-Cre mice; however, cleaved caspase-3 in Cd41+ tumor cells was significantly reduced in tumors from *Dicer1*^fl/fl^/*Pf4*-Cre mice (Fig 2B). We further characterized the miRNA enrichment of platelet-derived microvesicles from each genotype, by qRT-PCR for twelve selected mature miRNAs, established to be among the most enriched in platelets [55], from isolated microvesicles collected following *ex vivo* platelet stimulation. As shown in Fig 2C, platelet microvesicles were strongly depleted of these miRNAs compared with controls, consistent with similar miRNA depletion levels in the parent platelets [52]. Together, our results demonstrate that platelet miRNAs are drivers of primary tumor growth suppression in these solid tumor models.

### Platelet miRNAs broadly modulate mRNA expression in primary ectopic solid tumor cells in vivo

**Fig 3:**
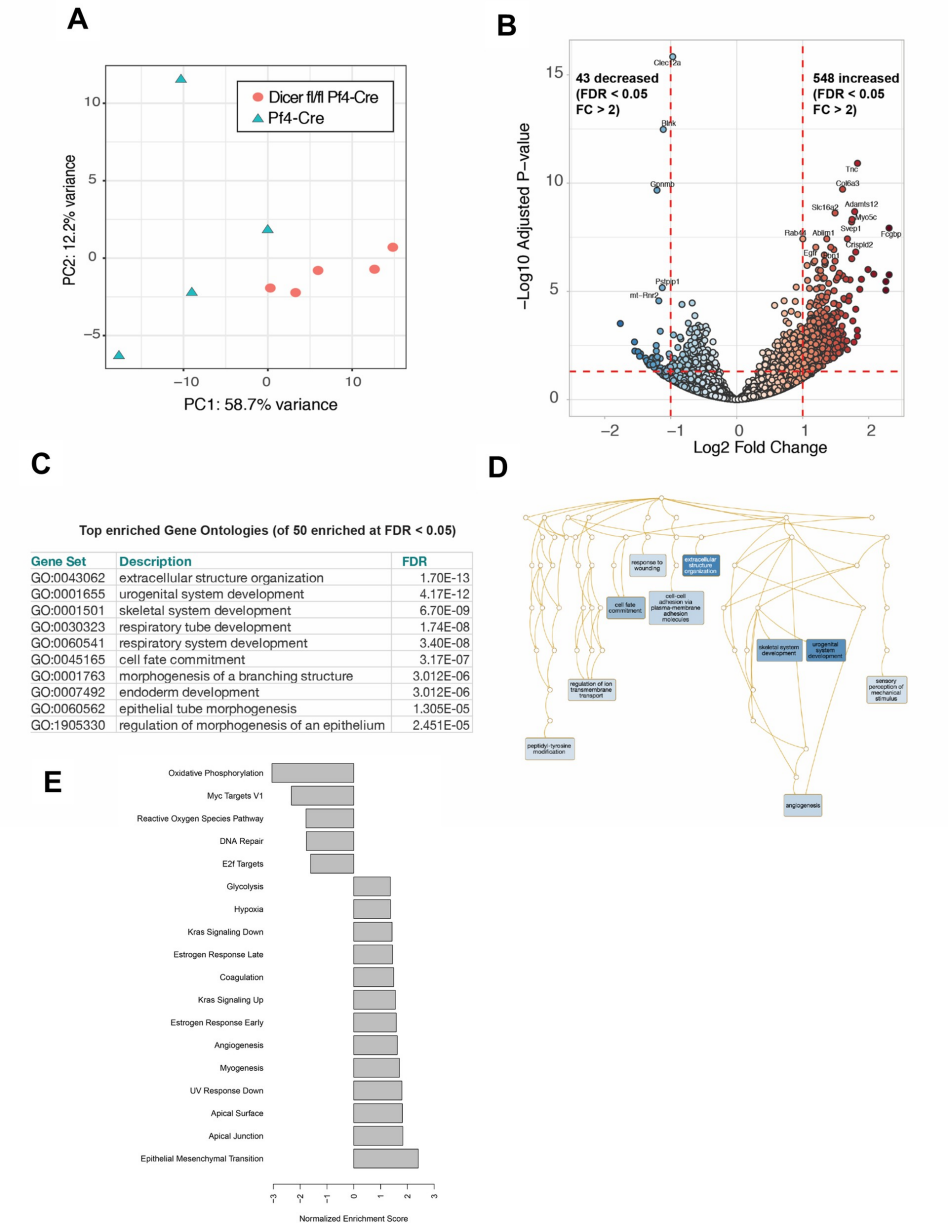
Modulation of tumor cell mRNA expression by platelet miRNAs in ectopic primary solid tumors *in vivo*. Tumors were resected, disrupted into single cell suspensions, the suspensions were labeled with Epcam antibodies, and tumor cells were single cell sorted for high Epcam-expressors as detailed in Methods (four control mice and five *Dicer1*^fl/fl^/*Pf4*-Cre mice). RNA was extracted and analyzed by RNA-seq for gene expression changes. **A** Principle component (PCA) analysis of global gene expression in tumors grown in *Dicer1*^fl/fl^/*Pf4*-Cre mice versus control mice. **B** Volcano plot of transcript changes between *Dicer1*^fl/fl^/*Pf4*-Cre mice versus control mice. Horizontal red line is set at -Log10 of adjusted P-val (FDR) = 0.05 (above the line are significant). Vertical red line set at log_(2)_ fold change of 2 (outside lines are larger fold change). **C** Top 10 gene ontologies determined by over representation analysis of the transcripts with FDR < 0.05 and fold change > 2. **D** The 50 significantly enriched gene ontology pathways were reduced into representative redundant ontologies using weighted set cover reduction. The relationship between the clustered pathways are plotted in directed acyclic graphs and are colored according to FDR (darker color is lower FDR). **E** Normalized enrichment scores for hallmark pathways, as detailed in S2 Table.

We next sought to determine the effects of platelet miRNA depletion on global mRNA expression in tumor cells in ectopic solid pancreatic cell tumors in mice. We resected tumors at 21d post-implantation, dispersed the resected tumors into single cell suspensions, and sorted carcinoma cells from the suspensions by flow-activated cell sorting using surface labeling with antibodies to Epcam, an epithelial-specific marker. Thus, sorted Epcam^+^ cell populations comprised carcinoma cells which had not undergone epithelial-mesenchymal transition, whereupon Epcam expression is typically lost [57]. We then extracted total RNA from the sorted tumor cells and subjected the RNA extracts to differential expression RNA sequencing (DEseq), following previous approaches for mRNA reads via poly(dT) oligomeric single end priming [52, 58]. Principle component analysis of transcriptome-wide gene expression across samples indicated broad modulation of mRNA expression in the sorted tumor cells as a function of platelet miRNAs in the host animals (Fig 3A). Using a fold change cutoff of 2 and an adjusted p-value (FDR) < 0.05, we observed significantly increased expression of 548 mRNAs in the tumor cells from platelet miRNA-depleted mice compared to controls, and significantly decreased expression of 43 mRNAs (Fig 3B and S1 Table). Changes included both increased and decreased mRNA expression in the tumor cells, but tilted strongly towards increased expression in the absence of miRNA, suggesting platelet miRNA suppression of tumor transcripts is relieved by Dicer1 deletion. However, we cannot deduce from these data whether changes to *in vivo* tumor cell mRNA expression mediated by platelet miRNAs were direct or indirect. As further evaluation of direct platelet miRNA effects on the tumor cell transcriptome, we performed miRNA target prediction analysis for the 548 increased mRNAs using miRNet (mirnet.ca), followed by filtering for the 44 most depleted miRNAs we previously identified in platelets from *Dicer1*^fl/fl^/*Pf4*-Cre mice compared to controls [52]. 42 miRNAs from this list matched to increased tumor cell mRNAs in this study, with 970 total matches together accounting for 321 out of the 548 increased tumor cell mRNAs (58.6%) harboring predicted or established miRNA recognition elements for these platelet miRNAs (S1 Table). Similarly, target prediction results filtered for the previously identified 50 miRNAs which are most highly enriched in human platelets (compiled from multiple studies in [55]) included 46 miRNAs from this list matched to increased tumor cell mRNAs, with 1658 total matches together accounting for 355 out of the 548 increased tumor cell mRNAs (64.8%) (S1 Table). Together, these data indicate that platelet-derived miRNAs broadly modulate tumor cell gene expression in solid tumors in this model.

Reflecting the broad effects of platelet miRNA depletion on the *in vivo* tumor cell transcriptome, significantly enriched gene ontology (GO) classifications of differentially expressed mRNAs covered a wide spectrum of biological processes. The top enriched ontologies ranked by FDR are shown in Fig 3C, with the full list of enriched GOs detailed in S2 Table. Redundancy reduction distilled these into representative ontologies with their relationships represented by the directed acyclic graph shown in Fig 3D, and normalized enrichment scores for the most significant hallmark pathways in Fig 3E. Notably, there were no significant changes in apoptotic effector Caspase family expression, indicating that reduction in enrichment for cleaved Caspase-3 in tumors from platelet miRNA-depleted mice (Fig 2) did not reflect overall reduction in total Caspase-3 expression in these tumor cells. Similarly, mRNA encoding Epcam, which we used as a surface marker to sort tumor cells from other cells in the tumor microenvironment, was not significantly altered between the two cohorts. Together, these DEseq results demonstrate that platelet miRNAs play broad modulatory roles in tumor cell mRNA expression in primary solid tumors.

**Fig 4:**
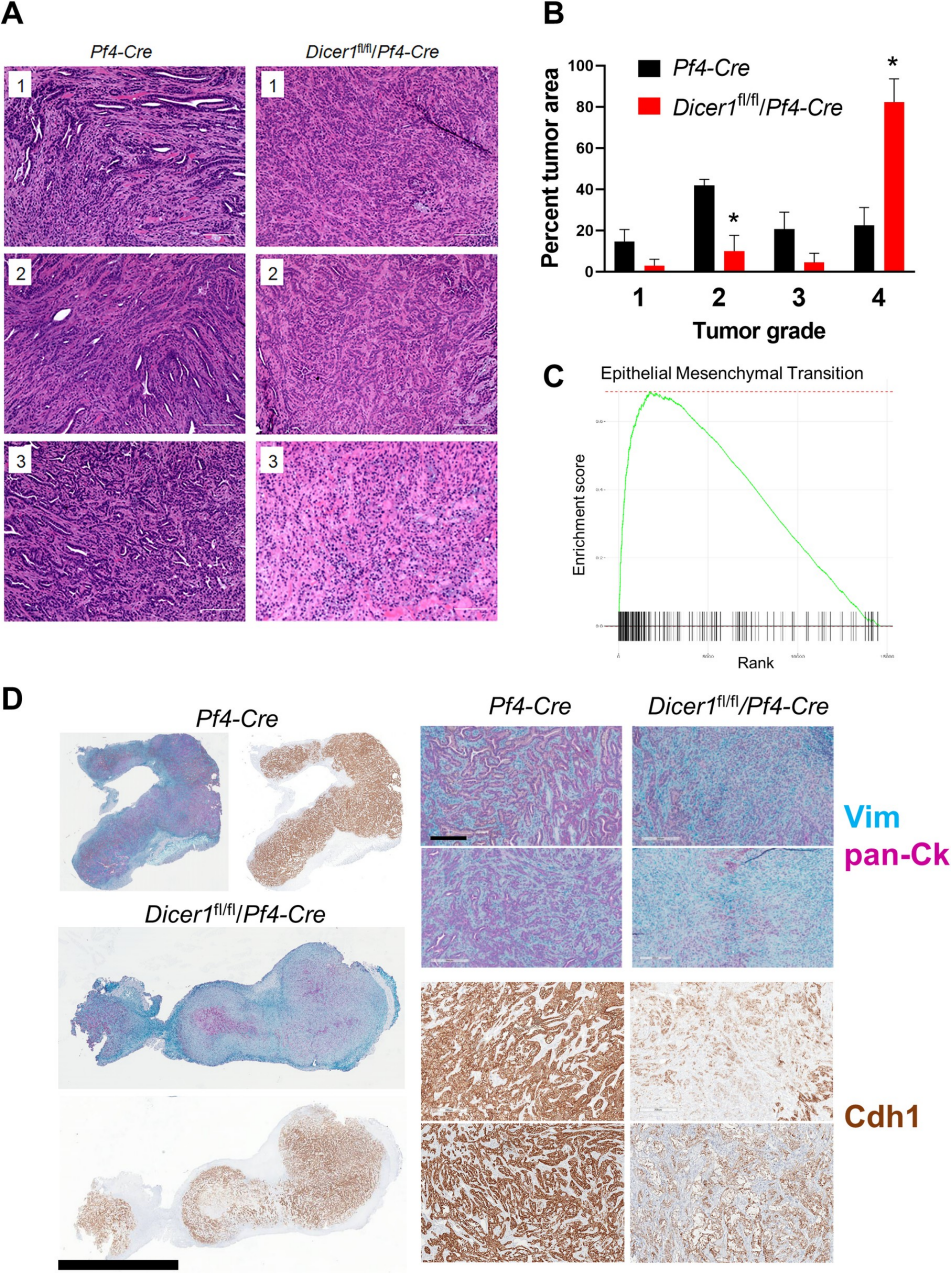
Increased tumor cell plasticity in ectopic tumors in platelet miRNA-depleted mice. Resected KPC primary tumors from *Pf4-Cre* or *Dicer1*^fl/fl^/*Pf4-Cre* mice were sectioned and processed for staining with hematoxylin and eosin, or staining for Cdh1, Ck and Vim. Whole slides were scanned for analysis. **A** Images (20x magnification) are representative of at least 5 separate image frames per tumor from 3 mice per genotype (#1–3 each). Bars, 200 μm. **B** Percent tumor area by grade from (**A**) + s.e.m. *n* = 3. *, *p* < 0.05. **C** Hallmark enrichment plot for Epithelial Mesenchymal Transition from **S2 Table**. **D** Whole tumor sections (left) and higher magnification views (right) from representative tumors stained with antibodies against Cdh1 (brown), pan-Ck (magenta) and Vim (teal). Representative images from central areas of 2 tumors against each background genotype are shown are shown to the right. Bars, left, 4 mm; right, 200 μm.

Interestingly, among the pathways and processes most strongly increased in the tumor cells in the absence of host platelet miRNAs were those related to epithelial-mesenchymal transition (EMT), cell-cell and cell-matrix junction interactions and organization (Figs 3E, 4C and S2 Table). Consistent with this observation, we observed increased areas of undifferentiated or sarcomatoid growth in the tumors derived from platelet miRNA-depleted mice compared to those from control mice (Fig 4A). Strikingly, over 80% of the tumor areas from platelet Dicer1-deleted mice were grade 4, compared with just over 11% grade 4 areas in tumors from control mice at the 21 day endpoint (Fig 4B). We tested this possibility further by staining tumor sections with antibodies to E-cadherin (Cdh1), Vimentin (Vim) and pan-Cytokeratins (pan-Ck) to assess EMT as a function of host platelet miRNAs. As shown in Fig 4D, Cdh1 and pan-Ck showed decreased staining in tumors resected from platelet miRNA-depleted mice compared to control mice, whereas Vim was increased. Together, these results indicate that modulation of tumor cell plasticity is a major mechanism of host platelet miRNA anti-tumor activity.

### Suppression of target protein expression by platelet miRNAs in tumor cells *in vivo*

**Fig 5:**
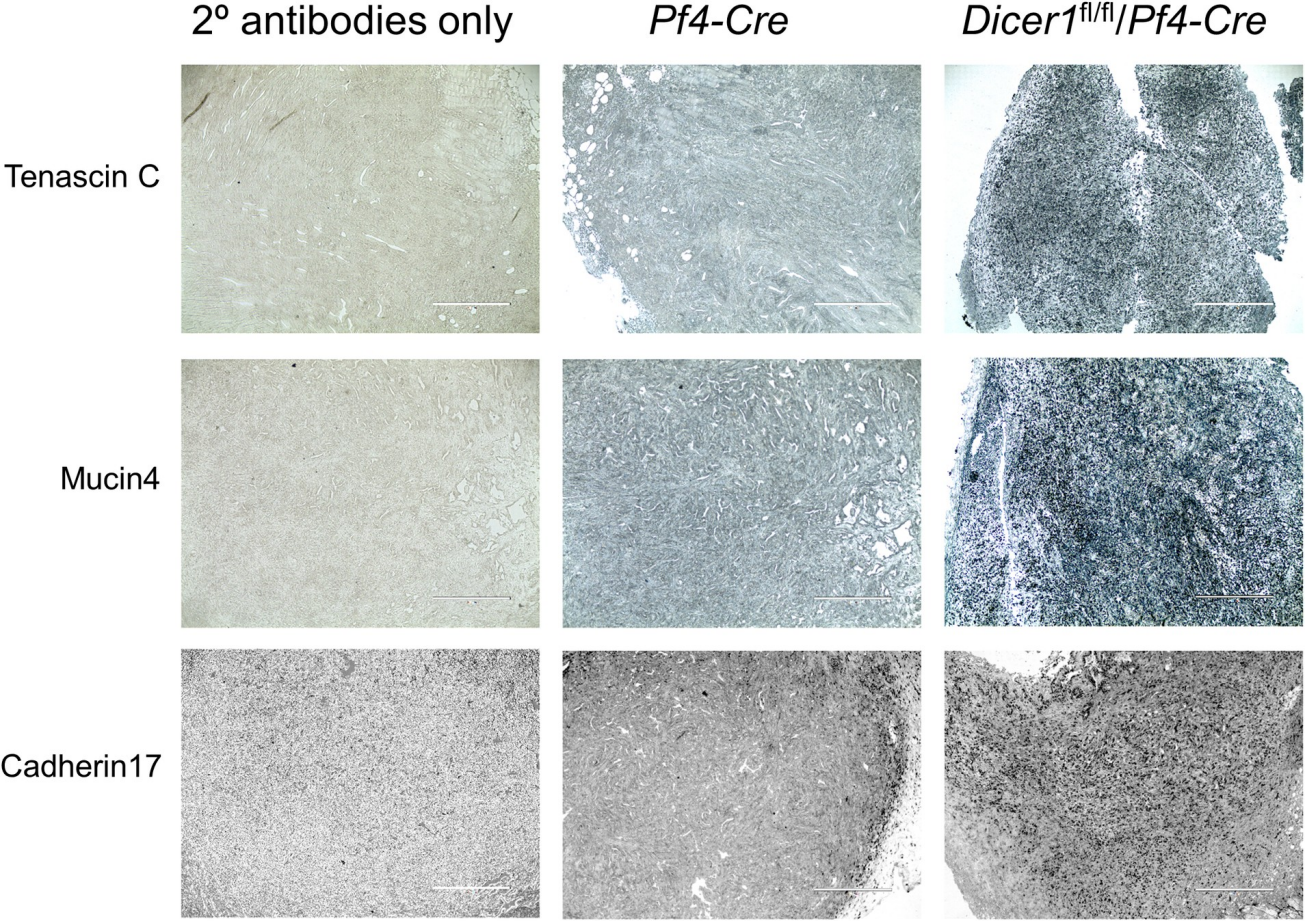
Suppression of protein expression of mRNA targets by platelet miRNAs in tumor cells *in vivo*. Resected KPC primary tumors from *Pf4-Cre* or *Dicer1*^fl/fl^/*Pf4-Cre* mice were sectioned and stained with the indicated antibodies or 2° antibody-only controls. Images (10x magnification) are representative of at least 5 separate image frames per tumor from 3 mice per genotype. Bars, 400 μm.

As further proof of concept that platelet miRNAs affect functional changes in tumor cell gene expression, we considered whether platelet miRNA-dependent changes to mRNA levels in the tumor cells, as indicated by RNA-seq, corresponded to altered protein expression in the tumors. To investigate this possibility, we stained slices of fixed and embedded tumor tissue from whole resected tumors with antibodies against three proteins we selected from among the most differentially expressed transcripts: Tenascin C (Tnc), Mucin 4 (Muc4), and Cadherin 17 (Cdh17). We selected these target proteins as each has an established role in pancreatic tumor progression [59–65]. Each of these proteins of interest showed substantial increased mRNA expression by DEseq in the tumor cells from tumors from *Dicer1*^fl/fl^/*Pf4-Cre* mice compared to *Pf4-Cre* mice: Tenascin C (log_(2)_ fold change 1.83 [3.55-fold increase], padj 1.22 x 10^−11^), Mucin 4 (log_(2)_ fold change 1.69 [3.22-fold increase], padj 2.76 x 10^−5^), and Cadherin 17 (log_(2)_ fold change 1.24 [2.36-fold increase], padj 0.0016) (S1 Table). As shown in Fig 5, we observed increased intratumoral staining indicating increased protein expression of Tenascin C, Mucin 4 and Cadherin 17 in tumors from *Dicer1*^fl/fl^/*Pf4-Cre* mice compared to *Pf4-Cre* mice. These data, focused on selected genes, indicate that platelet miRNAs can broadly modulate protein expression in tumor cells in solid tumors *in vivo*, associated with suppressed cognate mRNA expression and modulation of solid tumor growth.

**Fig 6:**
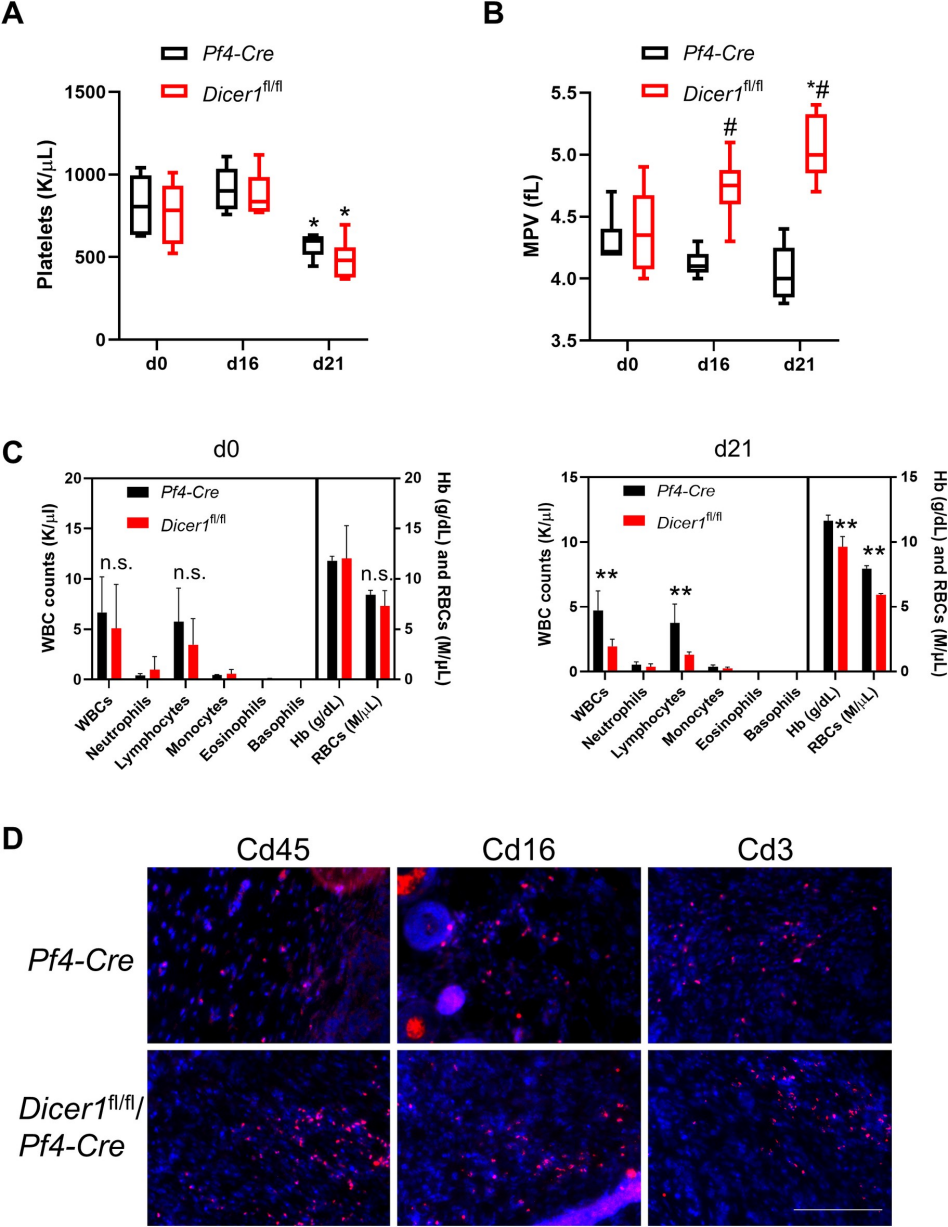
Peripheral blood cell counts, mean platelet volumes, and leukocyte/lymphocyte tumor infiltration in *Pf4-Cre* and *Dicer1*^fl/fl^/*Pf4-Cre* mice pre- and post-tumor implantation. Blood was sampled from mice by retroorbital bleeding at the indicated times pre- and post-implantation of KPC tumor cells. Peripheral blood (**A**) platelet counts, (**B**) mean platelet volumes (MPV), (**C**) leukocytes, erythrocytes and hemoglobin (Hb) were assessed by HEMAVET analysis of whole blood, and are shown ± s.e.m. Platelet box plots (**A**) show minimum, maximum, and mean counts. WBCs, white blood cells; RBCs, red blood cells. *n* = 6. *, *p* < 0.03 compared to the same genotype at d0; #, *p* < 0.001 compared to controls on the indicated days; **, *p* < 0.04 between genotypes; n.s., not significant. **D** Representative central images from d21 tumor sections immunostained with either Cd45, Cd16 or Cd3 as indicated (red), counterstained with DAPI to show nuclei. Bar, 200 μm.

With respect to the host animal platelets themselves, platelet counts and mean platelet volumes in peripheral blood were similar between the two groups prior to tumor implantation. Platelet counts were unaltered at d16, but by d21 of ectopic tumor growth, platelet counts were significantly decreased, but again to similar degrees in both control and *Dicer1*^fl/fl^/*Pf4*-Cre mice (Fig 6A). Interestingly, we observed increased mean platelet volumes in the tumor-bearing *Dicer1*^fl/fl^/*Pf4*-Cre mice, whereas platelets in tumor-bearing control mice showed a trend towards smaller platelet volumes, although this trend did not reach significance (Fig 6B). In contrast, lymphocytes were unexpectedly reduced in the *Dicer1*^fl/fl^/*Pf4*-Cre mice compared to controls at d21 of tumor growth. Red blood cell counts and hemoglobin concentration were also slightly reduced in the tumor-bearing *Dicer1*^fl/fl^/*Pf4*-Cre mice (Fig 6C). Following these results, we considered whether leukocyte/lymphocyte tumor infiltration may have been altered in the platelet miRNA-depleted mice. As shown in Fig 6D, distributions of leukocytes/lymphocytes, identified by Cd45/Cd16/Cd3 staining, appeared similar in the tumors in *Dicer1*^fl/fl^/*Pf4*-Cre and *Pf4*-Cre mice, indicating that the decreased levels of circulating leukocytes/lymphocytes did not alter their enrichment in the tumor microenvironment.

Total peripheral blood extracellular vesicles, observed by nanoparticle tracking analysis of platelet-poor plasma, increased substantially in tumor-bearing mice of both genotypes at d21, in particular large extracellular vesicles > 150 nm in diameter that likely include microvesicles (microparticles), but we did not observe significant differences in extracellular vesicle concentrations between *Pf4*-Cre and *Dicer1*^fl/fl^/*Pf4*-Cre tumor-bearing mice. Similarly, smaller particles, likely exosomes (particles < 150 nm in diameter) were not significantly different between the genotypes, and small extracellular vesicle/exosome concentrations were not altered after d21 of tumor growth (S1A Fig).

To evaluate platelet-derived microvesicles in tumor-bearing mice, we captured extracellular vesicles from platelet-poor plasma on poly-_L_-lysine-coated coverslips and stained with antibodies to Cd41, a platelet- and platelet microvesicle-specific marker [48, 68]. We counter-stained with antibodies to Tissue Factor (TF, also known as Factor III), a major determinant of thrombotic risk in pancreatic cancer and often associated with microvesicles in pancreatic cancer patients [66–79], as an indicator of the procoagulant nature of the microvesicle surfaces. Platelet-poor plasma from tumor-bearing mice was enriched with platelet microvesicles to a similar extent in both genotypes, and many of these microvesicles also displayed TF at their surfaces (S1B Fig). Together, these data indicate that the presence of ectopic pancreatic cancer cell primary tumors led to reduced platelet counts in these mice, procoagulant platelet microvesicles were increased in tumor-bearing mice, and these effects were independent of the host animal platelet miRNA background.

## Discussion

The results of this study demonstrate for the first time that host platelet-derived miRNAs potently modulate primary solid tumor growth *in vivo* through robust regulation of tumor cell gene expression. Accelerated ectopic tumor growth in animals lacking platelet miRNAs was associated with inhibition of apoptotic markers and increased expression of a broad spectrum of the tumor cell transcriptome, coupled with decreased expression of many other genes, pointing to a driving role of platelet miRNAs in controlling primary tumor growth by regulating the tumor cell transcriptome. The platelet miRNA-dependent tumor cell genes encompassed a range of pathways important for tumor growth, indicating that platelet miRNAs can modulate growth of tumor cells by interfering with multiple pathways. Together, our data demonstrate that platelet miRNAs are a significant regulatory component of platelet inhibitory roles in cancer progression at the stage of primary tumor growth, and we identify a broad panel of platelet miRNA-regulated genes in tumor cells in this ectopic tumor growth model. These findings may inform clinical perspectives on the platelet/cancer axis, such as putative effects of individual variance in miRNA expression or of anti-platelet drugs in control of tumor progression.

The principal finding of this study is that platelet miRNAs are sufficient to modulate growth of primary tumors in ectopic tumor growth models in mice. These results extend our previous findings which demonstrated that platelet microvesicles (PMV) harboring platelet-derived miRNAs transfer these miRNAs to tumor cells by infiltrating primary solid tumors and interacting directly with tumor cells, with tumor growth inhibitory effects [41]. While PMV are enriched in platelet-derived miRNAs, PMV also contain various proteins, lipids, second messengers, other RNA species including mRNAs, and even platelet mitochondria, confounding specific assignments for the roles of platelet miRNAs in regulation of tumor growth [42, 43, 80]. Moreover, as PMV release is enhanced by platelet activation which increases in the presence of cancer, it has been difficult to separate effects of PMV from those of activated platelets in tumor progression [81]. Our results demonstrate a specific and substantial role for platelet miRNAs *per se* as modulators of ectopic tumor growth, and this role was independent of the plasma PMV concentration which was unaltered by platelet miRNA depletion. This finding suggests that the enhanced tumor growth we observed by depletion of platelet miRNAs was not due to a difference in PMV generation or clearance, but in either the loss of miRNAs in PMV or platelet exosomes, or in platelets themselves. Indeed, the tumor modulatory effects of platelet miRNA depletion may reflect a combination of platelet-derived miRNA functions in tumor cells as a result of intracellular miRNA transfer, and in the originating platelets. We also noted the striking observation that mean platelet volumes were substantially increased in tumor-bearing mice on the platelet miRNA-depleted background, which may indicate increased production of younger, larger platelets, increased platelet protein expression causing larger platelet volumes, or possibly a balance of both. However, we observed reduced platelet counts at similar levels in mice of either genotype bearing advanced tumors compared to non-tumor-bearing mice, as expected due to enhanced platelet activation associated with disseminated intravascular coagulation which is common in the presence of cancer, particularly so for pancreatic cancer [82–86], suggesting that the overall balance of platelet activity leading to clearance *per se* was not drastically altered in this model. Platelets, and PMV, each play complex and as yet incompletely understood roles in cancer progression, notably with respect to immunomodulation that is an essential component of host anti-tumor response [26, 87–91]. Another important but unexpected finding of our study was that circulating lymphocyte counts were substantially suppressed in tumor-bearing platelet miRNA-depleted mice compared to tumor-bearing controls, whereas neutrophil and monocyte counts were similar. Tumor infiltration of leukocytes/lymphocytes was unaltered by host platelet miRNA deletion; however, we cannot completely rule out potential direct or indirect effects of *Pf4-Cre*-mediated Dicer1 deletion in blood cells in addition to platelets [92]. Thus, platelet miRNAs also modulate immune function in tumor-bearing mice that could have important impacts on the tumor microenvironment, underscoring a need for further study on this putative role of platelets in immunomodulation in cancer. Taken together, platelet miRNAs as a cohort are tumor suppressive in ectopic primary tumor growth, potentially via multiple mechanisms that demand further investigation.

In addition to putative effects of platelet miRNAs on other cells in the tumor microenvironment, platelet miRNA depletion had a substantial effect on the tumor cell transcriptome on a per cell basis, as evidenced by broad changes in poly(dT)-enriched RNAs from Epcam-sorted tumor cells from platelet miRNA-depleted mice compared to tumor cells from control mice. These data demonstrate a direct role for platelet miRNAs in modulating mRNA expression in primary tumor cells themselves. MiRNAs are generally considered as suppressors of mRNA expression, via multiple mechanisms that include target mRNA degradation initiated by Argonaute 2 within the RNA-induced silencing complex, which has been shown to be transferred as Argonaute 2-miRNA complexes from platelets to heterologous cells via PMV [25, 93]. Reflecting this general function of miRNAs, we observed significantly increased expression of 548 tumor cell mRNAs from the platelet miRNA-depleted mice, 321 of which harbor miRNA recognition elements for the 44 most depleted miRNAs in Dicer1-deleted platelets, indicating that platelet miRNAs are necessary to modulate expression of these genes. Conversely, we also observed down-regulation of 43 tumor cell mRNAs. While the overall numerical balance favors direct platelet miRNA-mediated tumor cell mRNA suppression by some 500+ genes, the down-regulated genes as a whole speak to a robust restructuring of the tumor cell transcriptome as a function of platelet miRNAs. At this stage we cannot separate direct and indirect effects of platelet miRNAs; hence, it is possible that certain genes were down-regulated as a result of up-regulation of other genes due to loss of suppressive platelet miRNAs. Indirect down-regulation of genes as a result of miRNA-mediated suppression of other genes regulating their steady state levels, such as transcription factors, is also possible. The particular miRNA:mRNA target species await direct mapping in future studies, which will help elucidate these effects. Moreover, some of the growth suppressive miRNAs and their mRNA targets are likely cancer type- or even cancer cell type-specific, while there may also be common pathways. The full cohort of platelet miRNA-regulated tumor cell mRNAs we mapped will be a valuable resource for dissecting the specific contributions of platelet miRNAs to tumor cell transcriptome regulation on a per gene basis for different cancer types.

The tumor cell RNAs that were up- and down-regulated as a function of platelet miRNAs *in vivo* comprised a wide range of cell growth signaling pathways, reflecting the broad-spectrum effects of depletion of the full cohort of platelet miRNAs. The most significantly up-regulated pathways included genes related to epithelial-mesenchymal transition and mediators of epithelial junctions (including Tenascin C, which we confirmed was up-regulated in the tumors), glycolysis, pro-angiogenic genes, and various growth signaling pathways including Wnt, transforming growth factor beta, Hedgehog, cytokine receptor signaling, and many others. The down-regulated genes also comprised various pathways, and interestingly these consisted in large part of pathways involved in transcription, gene splicing, and translation, as well as other pathways. Further studies will elucidate the roles of platelet miRNAs in modulating individual target genes and signaling families, and their specific effects on tumor progression. As noted above, altogether alterations in these growth pathways reflect the broad-spectrum restructuring of the tumor cell transcriptome, mapping to similar restructuring of the functional proteome, as a function of platelet-derived miRNAs, with the end result being a potentiation of growth of the tumor cells in the *in vivo* solid tumors. The complex interplay of up- and down-regulated pathways and their ultimate effects on tumor cell growth demand further investigation, including mapping the platelet miRNAs with their tumor cell target mRNAs.

## Conclusions

Platelet miRNAs as a cohort are major modulators of the growth of primary tumors, at least in these ectopic tumor growth models in mice. Identification of the key modulatory miRNAs will be critical to furthering our understanding of the overall mechanisms. We predict that the most abundant miRNAs in platelets—and in PMV which are increased by platelet activation in the presence of cancer—will account for the strongest gene suppressive effects, based on the 1:1 stoichiometric nature of miRNA:mRNA interactions [94]. It is considerably more difficult to predict how individual variance in platelet miRNA expression will associate with cancer progression, as the platelet/cancer axis is not only highly complex, but is most well understood to date in the context of platelets enhancing cancer morbidities such as by supporting tumor metastasis through various mechanisms, as well as in cancer-associated thrombosis [86, 95, 96]. The roles of platelet miRNAs in immunomodulation in cancer also demand further investigation. From a clinical perspective, the principal obstacle to deconstructing the roles of platelet miRNAs in human cancers is that patients most often present with symptoms not during initial growth of a primary tumor, but when cancer progression is well advanced. The results of this study advance the overall concept of platelet miRNAs as innate immune tumor growth suppressors in primary solid tumors. This concept adds new depth to the evolving roles of anti-platelet drugs, which also suppress PMV generation, in control of cancer progression, and conversely this may support development of miRNA delivery approaches with anti-tumor efficacy following further molecular mechanistic and physiological studies.

## Supporting information

S1 Fig Peripheral blood extracellular vesicles in *Pf4-Cre* and *Dicer1*^fl/fl^/*Pf4-Cre* mice pre- and post-tumor implantation. Blood was sampled from mice by retroorbital bleeding at the indicated times pre- and post-implantation of KPC tumor cells. **A** Total particles were counted in platelet-poor plasma by nanoparticle tracking. Representative histograms are shown. Counts are shown as particles/mL x 10^8^ with 1-nm bin diameters, *e*.*g*., size (nm) ± 0.5 nm. **B** Representative images of extracellular vesicles captured from equal volume fractions of platelet-poor plasma on poly-_L_-lysine-coated coverslips, then fixed and stained with antibodies to Cd41 (ref) and tissue factor (TF, green) as indicated. Overlap appears as yellow. Bar, 5 μm. *n* = 6.
(TIF)

S2 Fig Original uncropped and unadjusted images for western blot results in Fig 2A. The original, uncropped and unadjusted scanned blot images for cleaved Caspase-3 (**A**) and β-actin loading control (**B**) are shown.
(TIF)

S1 Table RNA-seq analysis of poly-A-isolated RNA in Epcam-sorted KPC tumor cells from d21 tumors resected from *Pf4-Cre* and *Dicer1*^fl/fl^/*Pf4-Cre* mice. Shown are the summary of total RNAs, mRNAs ranked by fold change, raw read counts, normalized read counts, rlog conversions, sample list, mRNAs by Ensembl ID, and predicted platelet miRNAs targeting the significantly increased mRNAs. Shown in the mRNAs ranged by fold change page (KO vs. WT) are Ensembl ID (A), gene symbol (B), Biotype (C) Chromosome (D), Gene description (E), Human homolog gene symbol (F), base Mean (G), log(2) fold change for KO (*Dicer1*^fl/fl^/*Pf4-Cre*) relative to WT (*Pf4-Cre*) (H), lfcSE (I), stat(J), raw p-value (K), and the log adjusted binomial p-value ranking statistic (L). Genes selected for protein expression analysis (**Fig 4**) are highlighted in yellow in the KO vs. WT table. ‘Dicer1 from Rowley et al’ shows the results of miRNet search for predicted miRNAs targeting the 548 significantly increased mRNAs in tumor cells (symbol, embl, entrez) in this study, then filtered for the top 44 miRNAs depleted in platelets from *Dicer1*^fl/fl^/*Pf4-Cre* mice compared to controls [52]. Search criteria were the list of mRNA embl IDs, and the selected species was *Mus musculus*. ‘miR from Sunderland’ shows the results of miRNet search for predicted miRNAs, filtered for the top 50 miRNAs enriched in human platelets as compiled in [55], targeting the 548 significantly increased mRNAs in tumor cells (symbol, embl, entrez) in this study. Search criteria were the list of mRNA embl IDs, and the selected species was *Mus musculus*.
(XLSX)

S2 Table Pathway analysis from RNA-seq data (S1 Table) of poly-A-isolated RNA in Epcam-sorted KPC tumor cells from d21 tumors resected from *Pf4-Cre* and *Dicer1*^fl/fl^/*Pf4-Cre* mice. Shown are major pathways significantly altered in DEseq analysis between tumor cells from resected tumors from *Pf4-Cre* and *Dicer1*^fl/fl^/*Pf4-Cre* mice: cancer hallmark pathways, KEGG pathways, reactome, and gene ontology (GO) classifications by biological processes (BP), molecular function (MF), and cellular component (CC).
(XLSX)
