# Supplementary figures and images for: Platelet microRNAs inhibit primary tumor growth via broad modulation of tumor cell mRNA expression in ectopic pancreatic cancer in mice
Source: PLoS One. 2021 Dec 22;16(12):e0261633. doi: 10.1371/journal.pone.0261633 (PMC8694476; doi:10.1371/journal.pone.0261633)

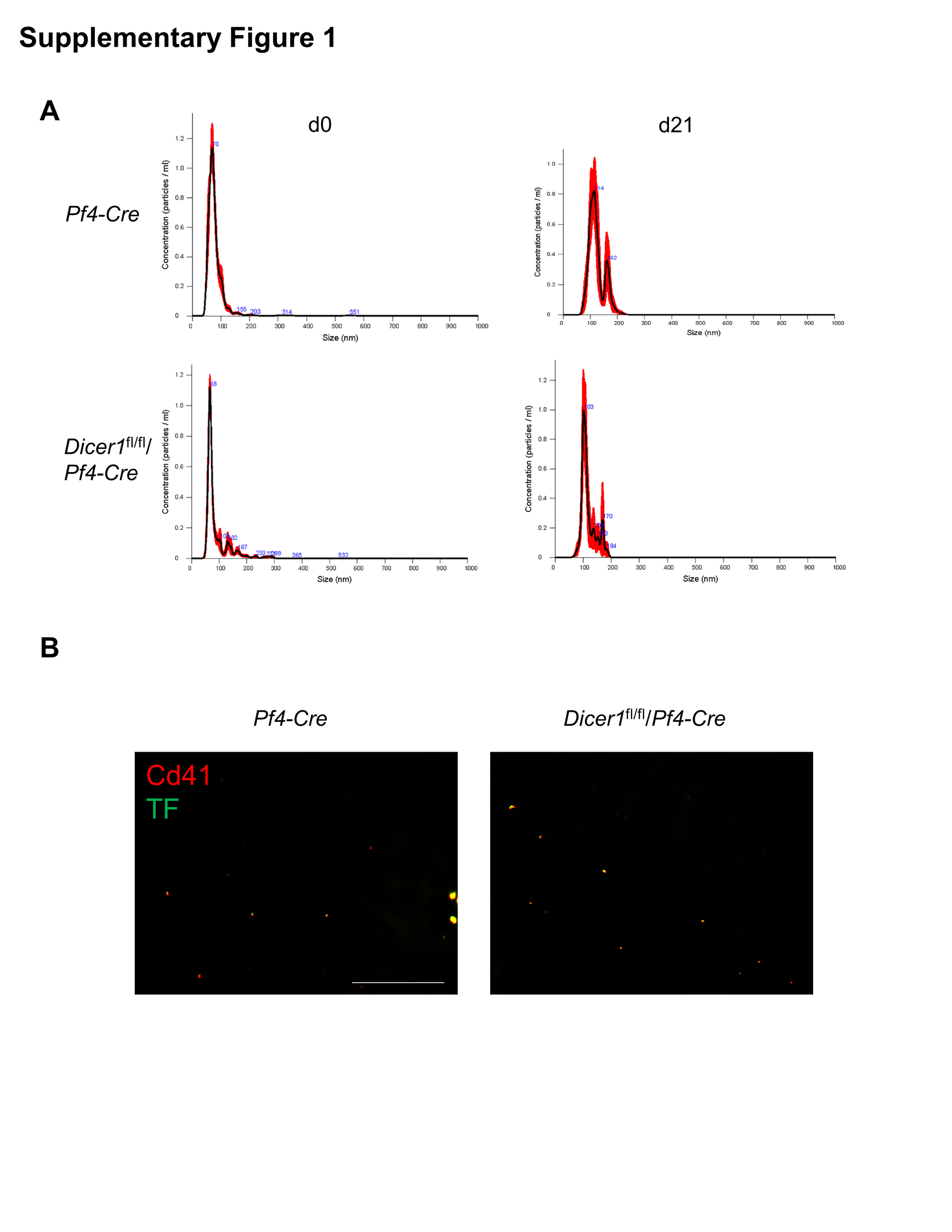

Supplement: S1 Fig — Blood was sampled from mice by retroorbital bleeding at the indicated times pre- and post-implantation of KPC tumor cells. A Total particles were counted in platelet-poor plasma by nanoparticle tracking. Representative histograms are shown. Counts are shown as particles/mL x 108 with 1-nm bin diameters, e.g., size (nm) ± 0.5 nm. B Representative images of extracellular vesicles captured from equal volume fractions of platelet-poor plasma on poly-L-lysine-coated coverslips, then fixed and stained with antibodies to Cd41 (ref) and tissue factor (TF, green) as indicated. Overlap appears as yellow. Bar, 5 μm. n = 6. (TIF) [file pone.0261633.s001.tif]

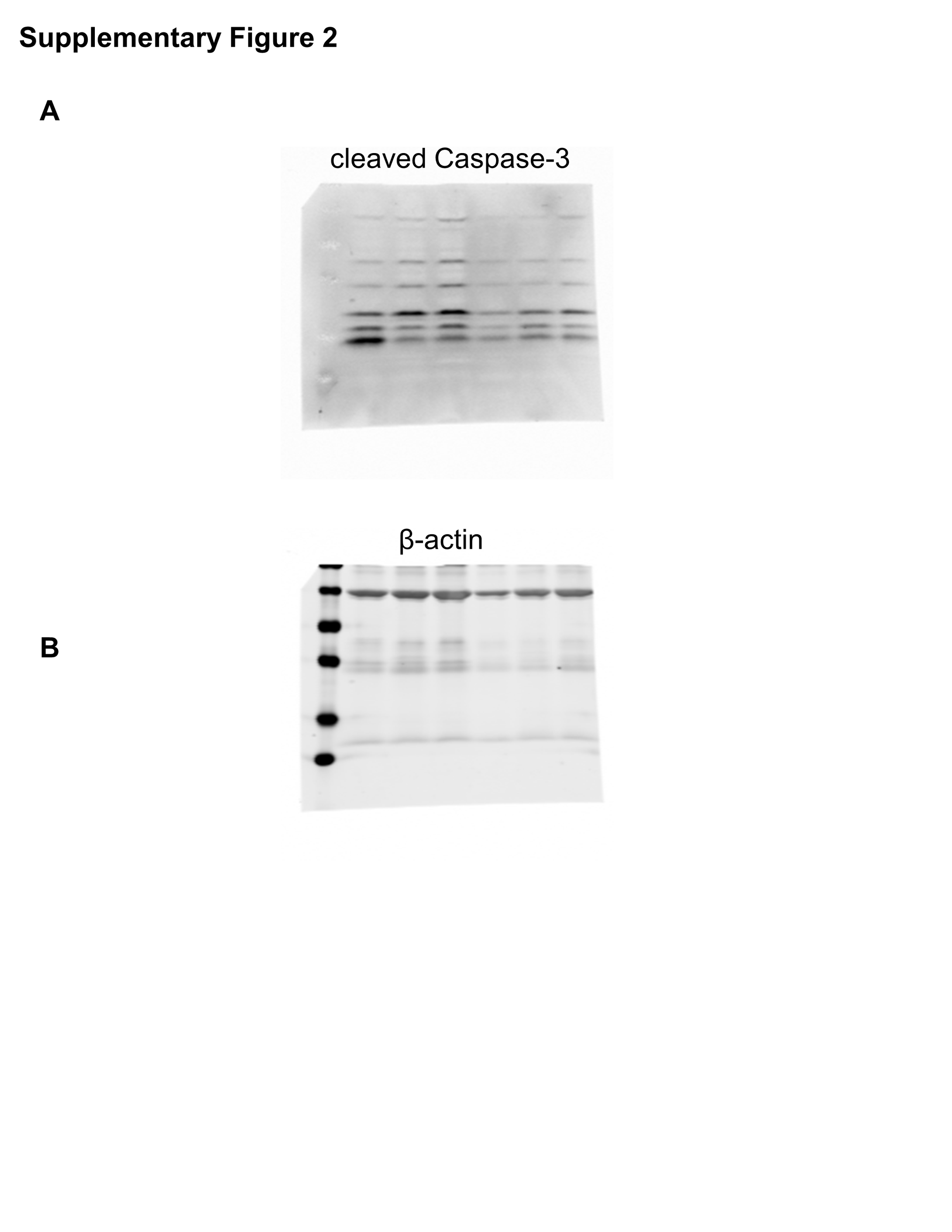

Supplement: S2 Fig — The original, uncropped and unadjusted scanned blot images for cleaved Caspase-3 (A) and β-actin loading control (B) are shown. (TIF) [file pone.0261633.s002.tif]
